# Optimization of Ursolic Acid Extraction in Oil from Annurca Apple to Obtain Oleolytes with Potential Cosmeceutical Application

**DOI:** 10.3390/antiox12020224

**Published:** 2023-01-18

**Authors:** Maria Maisto, Vincenzo Piccolo, Ettore Novellino, Elisabetta Schiano, Fortuna Iannuzzo, Roberto Ciampaglia, Vincenzo Summa, Gian Carlo Tenore

**Affiliations:** 1 Department of Pharmacy, University of Naples Federico II, via Domenico Montesano 49, 80131 Naples, Italy; 2 Faculty of Medicine, University Cattolica del Sacro Cuore, Largo Francesco Vito, 00168 Rome, Italy

**Keywords:** ursolic acid, oleolyte, antiaging, response surface methodology, biocompatible solvent

## Abstract

Ursolic acid (UA) is a plant-derived molecule with relevant anti-aging activity, which makes this molecule a potential functional active ingredient in cosmetic formulations. The main objectives of this study were to optimize the UA extraction process from Annurca apple (AA) with sunflower oil as a lyophilic food-grade solvent using Response Surface Methodology (RSM) to determine the potential cosmetic application of the obtained extract. The results of RSM analysis showed a maximum UA yield of 784.40 ± 7.579 (μg/mL) obtained under the following optimized conditions: sunflower oil as extraction solvent, 68.85 °C as extraction temperature, and 63 h as extraction time. The HPLC-DAD-HESI-MS/MS analysis performed on the extract obtained under these conditions, named Optimized Annurca Apple Oleolyte (OAAO), led to the identification of twenty-three phenolic and terpenoid molecules and the quantification of eight of them. To explore the biological properties of OAAO, the in vitro antioxidant activity was evaluated by DPPH, ABTS, and FRAP assays, resulting in 16.63 ± 0.22, 5.90 ± 0.49, and 21.72 ± 0.68 μmol Trolox equivalent/g extract, respectively. Moreover, the permeation study has shown that OAAO may be considered a safe and functional ingredient in potential cosmetic formulations.

## 1. Introduction

Ursolic acid (UA) (3β-hydroxy-urs-12-en-28-oic-acid) is a pentacyclic triterpenoid carboxylic molecule widely distributed in herbs, leaves, flowers, and fruits [1]. In natural matrices, this molecule may occur in free, in saponin-complexed form, or in its structural isomer (different substitution of methyl group), that is, oleanolic acid (3β-hydroxy-olea-12-en-28-olic-acid; OA) [1]. In recent years, UA and its derivatives have attracted considerable attention due to their functional properties, such as antioxidant [1], antitumor [1], anti-inflammatory [1], and antibacterial activities [2]. More specifically, great attention has been paid to the potential cosmetic application of UA, in line with the increasing trend of valorizing plant molecules as bioactive agents in cosmetic formulations [3,4,5,6]. Different studies conducted on human keratinocytes have shown a valuable increase in ceramide production after UA treatment [7]. It is well documented that the reduction in epidermal ceramide content is the main cause of impaired epidermal barrier function, leading to sensitive skin, increased transepidermal water loss, and high skin susceptibility to irritant agents [1]. These findings have also been confirmed by human studies, in which the recovery of epidermal permeability barrier function was improved after topical application of UA, as well as the ceramide content in the epidermis [7]. Moreover, UA treatment of human fibroblasts has shown a significant increase in collagen content [7,8]. In addition, UA has also been used as a functional agent in topical formulations for its broad cosmetic bioactivity, e.g., to improve and prevent skin roughness [9] and acne [10], and as a skin whitening agent due to its capacity to inhibit tyrosinase activity [11]. Since the reduction of dermal collagen content and skin dehydration have been commonly defined as the main cause of skin wrinkles and xerosis, UA can be recognized as a novel and natural anti-aging agent suitable for the development of cosmeceutical formulations.

The main natural UA sources are medicinal plants (mainly those belonging to the *Lamiaceae* family, such as *Rosmarinus officinalis),* flowers, and leaves of a wide variety of plant species. The recent literature has reported UA concentration in edible fruits, such as valuable levels in argan fruits (1.08 mg/g dw) [12], hawthorn (1.14 mg dw) [13], cranberry (7.09 mg/g dw) [14], jujube (0.53 mg/g dw) [15] and apple peel (14.3 mg/g dw) [1,16]. Annurca apple is the only apple cultivar native to southern Italy, listed as a Protected Geographical Indication (PGI) product by the European Council [Commission Regulation (EC) No. 417/2006)]. The nutraceutical potential of Annurca polyphenols has been largely documented for its beneficial effects on the management and control of cholesterol plasma levels in healthy and mildly hypercholesterolemic patients [17,18,19,20]. Specifically, UA and its peculiar structural isomer Annurcoic acid (subtraction with hydroxyl group), isolated exclusively in this apple cultivar, has been defined as the main triterpenoid components of the Annurca apple [21]. Due to the lipophilic nature of UA, ethyl acetate, methanol, petroleum ether, chloroform, and ethanol are commonly used for extraction from natural sources [22,23]. Among the above-mentioned solvents, only ethanol is regarded as suitable for obtaining extracts compatible with human health. Nevertheless, its use remains limited due to its high cost. Such considerations are in line with the current search for innovative extraction protocols of UA, involving alternative solvents, safe, possibly from natural sources and of low cost.

In light of what is stated above, the main objective of the present study was to investigate the potential of lyophilized Annurca Apple (AA) as a source of triterpenic acids, especially ursolic acid, for the development of potential natural cosmetic formulations. To optimize the UA extraction conditions from AA, the response surface methodology (RSM) was applied in order to assess the maximum yield of ursolic acid using sunflower oil as a food-grade lipolytic extraction solvent. The extract obtained under optimized conditions (OAAO, Optimized Annurca Apple Oleolyte) was qualitatively characterized by HPLC-DAD-HESI-MS/MS and quantitatively quantified by HPLC-DAD analysis, which was opportunely validated in the current work. Finally, its potential in vitro antioxidant activity was evaluated, and to assess its potential use as a functional ingredient in cosmetic formulations, a classical in vitro Franz cell experiment was performed to investigate the skin penetration behavior of UA.

## 2. Materials and Methods

### 2.1. Reagents

All chemicals, reagents, and standards used were analytical or LC-MS grade reagents. The water was treated in a Milli-Q water purification system (Millipore, Bedford, Burlington, MA, USA) before use. Sunflower oil was purchased in a local market. Ursolic acid (purity ≥ 98.5% HPLC), rutin (purity ≥ 94% HPLC), quercetin 3-O-glucoside (purity ≥ 98% HPLC), kaempferol 3-O-glucoside (purity ≥ 90% HPLC), kaempferol 3-O-rhamnoside (purity ≥ 98% HPLC), phloridzin (purity ≥ 99% HPLC), phloretin (purity ≥ 98.5% HPLC), gallic acid (purity ≥ 99% HPLC), 6-hydroxy-2, 5, 7, 8-tetramethylchromane-2-carboxylic acid (purity > 97% HPLC) were purchased from Sigma-Aldrich (Milan, Italy).

### 2.2. Sample Collection and Oleolyte Preparation

#### 2.2.1. Oleolyte Preparation Protocol and Experimental Design

Annurca apple fruits (Malus pumila Miller cv Annurca; about 100 g each) were collected in Valle di Maddaloni (Caserta, Italy) in October 2021 when the fruits had just been harvested (green peel). The fruits were reddened, following the typical treatment for about 30 days [24], and then analyzed. After this time, the apples were washed and sliced for freeze-drying. The oleolyte preparation was performed by adding a defined weight of AA to a certain volume of a deacidified sunflower oil (g/mL) in a ratio of 1:4. At the end of the matrix maceration in oil, the mixture was centrifuged at 9000 rpm for 10 min. The oil supernatant was collected and stored protected from light at 4 °C until analysis. As reported in Table 1, different extraction times (1, 2, 4, 12, 24, 48, and 96 h) and incubation temperatures (20, 40, 60, and 80 °C) were opportunely combined to optimize the ursolic acid incorporation in the oleolyte. All optimization conditions were performed in triplicate.

#### 2.2.2. Oil Deacidification Procedure

The oil was deacidified by performing a liquid-liquid extraction with an alkaline solution. A volume of 250 mL of a sodium carbonate solution (Na_2_CO_3_ 7.5%, *w*/*v*) was mixed with 250 mL of n-hexane and added with 500 mL of sunflower oil [25]. The mixture was stirred for 10 min, and the organic phase was separated by liquid-liquid extraction. The organic phase was washed with 1000 mL of water to remove the traces of the alkaline solution. The mixture was left under stirring for 10 min, and the organic phase was recovered by liquid-liquid extraction. Finally, the hexane was evaporated under a vacuum at 35 °C to obtain a deacidified oil.

#### 2.2.3. Acidity Determination of Oil Samples

The determination of acidity, expressed as the weight percentage of oleic acid, was performed in agreement with Regulation (EU) No 2016/1227. A mixture of diethyl ether and ethanol (50:50 *v*/*v*) was neutralized with a solution of potassium hydroxide 0.1 M, with 300 µL of an ethanolic solution of phenolphthalein 0.03 M added. An aliquot of oil (2.5 g) was dissolved in 50 mL of the neutralized solvent mixture. The mixture was titrated whit stirring with an aqueous solution of potassium hydroxide 0.1 M until the color change of the pH indicator. All determinations were performed in triplicate. The acidity was calculated according to the following formula: (V × c × M)/(10 × m), where V is the volume (ml) of the titrated potassium hydroxide solution, c is the concentration (M) of titrated potassium hydroxide solution, M is the molar mass in grams per mole of oleic acid (282 g/mol), and m is the mass (g) of the oil sample.

### 2.3. Triterpenoic Analysis

#### 2.3.1. Ursolic Acid Extraction Protocol

A volume of 120 mL of a solution of sodium carbonate Na_2_CO_3_ 7.5% (*w/v*) was added to 60 mL of OAAO. The mixture was stirred for 10 min, and the aqueous phase was separated by liquid-liquid extraction. The aqueous phase was acidified with 2 N hydrochloride acid at pH = 3, frozen and lyophilized. To the solid residue, a volume of 20 mL of ethyl acetate was added. The mixture was vortexed for 1 min and placed in an ultrasonic bath (Branson Fisher Scientific 150 E Sonic Dismembrator) for 10 min. Samples were then shaken on an orbital shaker (Sko-DXL, Argolab, Carpi, Italy) at 600 rpm for 10 min and centrifuged at 9000 rpm for 10 min. The supernatants were collected and stored at 4 °C protected from the light. The obtained pellets were re-extracted with 10 mL of ethyl acetate using the same procedure. Finally, the extracted obtained were evaporated to dryness under a light stream of nitrogen, reconstituted in dimethylsulfoxide (DMSO) at a concentration of 30 mg/mL, diluted with acetonitrile at a concentration of 5 mg/mL, and stored at −20 °C until analysis.

#### 2.3.2. Ursolic Acid Quantitative Analysis by HPLC-DAD

A Jasco Extrema LC-4000 HPLC system (Jasco Inc., Easton, MD, USA), coupled with an autosampler, a binary solvent pump, and a diode-array detector (DAD), was used for the analysis. Separation was performed according to the previously described method with slight modifications [26]. The column used was a Kinetex^®^ C18 column (250 mm × 4.6 mm, 5 μm; Phenomenex, Torrance, CA, USA). Water with 0.1% formic acid (A) and acetonitrile (B) were used as mobile phases. The elution gradient was performed under the following conditions: 0–3 min, isocratic on 60% phase B; 3–20 min, linear gradient from 60 to 90% B; 20–24 min, isocratic with 90% B; 24–29 min, isocratic on 60% B for column reconditioning. The injection volume was 20 µL, the column temperature was set at 30 °C, and the flow rate was set at 1 mL/min. The quantification of ursolic acid was performed at 205 nm [26].

#### 2.3.3. Linearity and Sensitivity of the Ursolic Acid HPLC-DAD Analysis

An analytical standard of ursolic acid was used to develop and validate the HPLC-DAD method used to evaluate the UA title in each extraction performed. A stock solution of the UA standard was prepared at a concentration of 1000 ppm using HPLC-grade acetonitrile as solvent. Six different concentrations (0.001, 0.005, 0.01, 0.05, 0.1, and 0.5 mg/mL) were prepared from the standard stock solutions and analyzed by HPLC in triplicate. A 6-point calibration curve was constructed by plotting the peak area against the standard concentration to evaluate the linearity of the method. Limits of detection (LODs) and limits of quantification (LOQs) were determined to evaluate the sensitivity of the method. Determination of the signal-to-noise ratio is performed by comparing measured signals from samples with known low concentrations of analyte with those of blank samples and previously described [27,28] LODs establishing the minimum concentration at which the analyte can be reliably detected as is defined as the lowest detectable concentration of analyst that the analytical system can reliably distinguish from the background level (S (signal of compound)/N (signal of noise)) = 3, while LOQ is defined as the lowest quantifiable concentration of analyst that can be measured with a standard level of confidence, and it is typically calculated using (S/N) = 10 [27,28].

#### 2.3.4. Accuracy and Precision of Ursolic Acid HPLC-DAD Analysis

As recommended by the ICH guidelines, to validate an analytical method, it is essential to determine the accuracy (estimated by calculating the % bias) and precision (estimated by calculating the % CV, coefficient of variation %) of the developed method [28]. Accuracy (% bias) was calculated by intraday and inter-day analysis of calibration standards. Three different UA concentrations were injected 3 times per day (intra-day) and once for 3 consecutive days (inter-day). Precision (%CV, coefficient of variation %) was determined by an intraday and inter-day analysis of UA calibration standards at 3 different concentrations. Each analyte was injected 3 times per day (intra-day) and once for 3 consecutive days (inter-day).

#### 2.3.5. Matrix Effect of Ursolic Acid Extraction

The matrix effect was investigated by calculating the ratio of the peak area in the presence of matrix (matrix spiked with Ursolic acid post extraction) to the peak area in the absence of matrix (Ursolic acid in acetonitrile). The matrix was spiked with the analyte in triplicate with 10 µg (low), 20 µg (medium), and 30 µg (high). The ratio was calculated as follows:

$$\mathrm{Matrix}\mathrm{effect}\;\%=\frac{\mathrm{Peak}\;\mathrm{area}\;\mathrm{in}\;\mathrm{presence}\;\mathrm{of}\;\mathrm{matrix}\;}{\mathrm{Peak}\;\mathrm{area}\;\mathrm{in}\;\mathrm{solvent}}\;\cdot 100$$

#### 2.3.6. Recovery of Ursolic Acid Extraction

The matrix effect was investigated by calculating the ratio of the peak area in the pre-extraction spiked samples (matrix spiked with Ursolic acid pre-extraction) to the peak area in the post-extraction spiked samples (matrix spiked with Ursolic acid post-extraction). The matrix was spiked with the analyte in triplicate with 10 µg (low), 20 µg (medium), and 30 µg (high) either before (pre-extraction spiked) or after (post-extraction spiked) extraction. The ratio was calculated as follows:

$$Recovery\;\%=\frac{{\mathrm{Peak}\;\mathrm{area}}_{pre-extraction\;spiked\;sample}\;}{{\mathrm{Peak}\;\mathrm{area}}_{post-extraction\;spiked\;sample}}\;\cdot 100\;$$

### 2.4. Polyphenols Analysis

#### 2.4.1. Polyphenolic Extraction

An aliquot of OAAO (8 g) was dissolved in n-hexane (8 mL) and mixed with 8 mL of a solution of 80% methanol with 1% formic acid [29], shaken for 10 min on an orbital shaker (Sko-DXL, Argolab, Carpi, Italy) at 600 rpm. The hydroalcoholic phase was separated with a liquid-liquid separation and protected from light at 4 °C. The oil phase was re-extracted with the same procedure using 8 mL of the hydroalcoholic mixture. Finally, the extracts obtained were evaporated to dryness under a gentle stream of nitrogen, reconstituted in a hydroalcoholic mixture at a concentration of 20 mg/mL, and stored at −20 °C until analysis of maintenance.

#### 2.4.2. Polyphenolic Quantitative Analysis by HPLC-DAD Analysis

Polyphenols quantitative analysis of the OAAO hydroalcoholic fraction was performed with an HPLC Jasco Extrema LC-4000 system (Jasco Inc., Easton, MD, USA) equipped with an autosampler, a binary solvent pump, and a diode-array detector (DAD) validated method [27]. Chromatographic analysis was performed according to our previously developed, and Chalcones were monitored at 280 nm, while flavonols were monitored at 360 nm. The mobile phases were water at 2% formic acid (solvent A) and a solution at 0.5% formic acid in acetonitrile and water 50:50, *v*/*v* (solvent B). Separation was performed using as column a Kinetex^®^ C18 column (250 mm × 4.6 mm, 5 µm; Phenomenex, Torrance, CA, USA): 0–5 min of 10% (B), from 10% (B) to 55% (B) in 50 min and 95% (B) in 10 min, followed by 5 min. The injection volume was 20 µL, the column temperature was set at 30 °C, and the flow rate was set at 1 mL/min. Peak identification was based on a comparison of retention times with analytical standards and standard addition to the samples. Quantitative analyses were performed using the calibration curve calculated with 6 different concentrations in a concentration range of 0.1–1000 ppm and triplicate injections at each concentration level.

### 2.5. OAAO Qualitative Composition by HPLC-DAD-HESI-MS/MS Analysis

An HPLC DIONEX UltiMate 3000 (Thermo Fisher Scientific, San Jose, CA, USA) equipment, coupled with an autosampler, a binary solvent pump, a diode-array detector (DAD), and an LTQ XL mass spectrometer (Thermo Fisher Scientific, San Jose, CA, USA), were used for the analysis. Separation was performed using as column a Kinetex^®^ C18 column (75 mm × 2.1 mm, 2.6 µm; Phenomenex, Torrance, CA, USA). The mobile phases were water at 0.1% formic acid (A) and acetonitrile at 0.1% formic acid (B). Elution was performed according to the following conditions: 0–3 min hold at 5% solvent B, from 5% (B) to 95% (B) in 22 min, followed by 3 min of maintenance; for the remaining 3 min, the column was equilibrated to the initial conditions. The injection volume was 5 µL, the column temperature was set at 35 °C, and the flow rate was set at 0.35 mL/min. For the mass parameters, the source was a heated electrospray interface (HESI) operated in negative ionization with full scanning (FS) and data-dependent acquisition (DDA). Chalcones were monitored at 280 nm, while flavonols were monitored at 360 nm. Collision-induced fragmentation was made using argon, with a collision energy of 35.0 eV. The source operated in negative ionization mode for the analysis of the polyphenolic extract and both in positive and negative ionization modes for the analysis of the triterpenoic extract. The ion source was set for positive ionization mode using the following parameters: auxiliary gas flow rate: 10; sheath gas flow rate: 30; source heated temperature: 150 °C; capillary temperature: 320 °C; source current: 100 µA; source voltage: 3.5 kV; tube lens: 80 V; and capillary voltage: 32 V. The ion source was set for negative ionization mode using the following parameters: auxiliary gas flow rate: 10; sheath gas flow rate: 30; source heated temperature: 150 °C; capillary temperature: 320 °C; source current: 100 µA; source voltage: 3.5 kV; tube lens: 90 V; and capillary voltage: 31 V.

### 2.6. Total Phenolic Content Determination

The total phenol content (TPC) was performed by Folin–Ciocalteau’s assay, using gallic acid as the reference standard (Sigma-Aldrich, St. Louis, MO, USA) [30]. Briefly, 0.1 mL of the samples were diluted with water to obtain an absorbance value included in the linear range of the spectrophotometer. The reactives were added to the samples sequentially: 0.5 mL of Folin–Ciocalteau’s (Sigma-Aldrich, St. Louis, MO, USA) reagent and 0.2 mL of an aqueous solution of Na_2_CO_3_ 7% (*w/v*), bringing the final volume to 10 mL with water. Then, the samples were shaken and incubated for 90 min in the dark. After the reaction time, the absorbance was measured at 760 nm (Jasco Inc., Easton, MD, USA). The analysis was performed in triplicate, and the total polyphenols concentration was expressed in gallic acid equivalents (GAEs).

### 2.7. Antioxidant Activity

#### 2.7.1. DPPH^•^ Radical Scavenging Assay

The radical scavenging ability of the antioxidants in the sample was evaluated using the stable radical 2,2-diphenyl-1-picrylhydrazyl (DPPH) [31]. The analysis was performed by mixing 100 µL of each sample, opportunely diluted in an extraction mixture, with 1000 µL of a DPPH methanolic solution (153 mmol/L). The mixture was left in incubation for 10 min of reaction time in the dark. The decrease in absorbance was evaluated using a UV–visible spectrophotometer (Beckman, Los Angeles, CA, USA). After the reaction time, the absorbance was measured at 517 nm. All determinations were performed in triplicate. DPPH^•^ inhibition was calculated according to the formula: [(A_i_ − A_f_ )/A_c_] × 100, where A_i_ is the absorbance of the sample at t = 0, A_f_ is the absorbance of the sample after the reaction time, and A_c_ is the absorbance of the control, obtained by mixing 1000 µL of a DPPH methanolic solution with 100 µL of methanol. The obtained results are expressed in µmol of Trolox (6-hydroxy-2, 5, 7, 8-tetramethylchroman-2-carboxylic acid) equivalent (TE). Moreover, the results were also reported as EC_50_, which is the amount of antioxidant compound necessary to neutralize the initial DPPH^•^ concentration by 50%.

#### 2.7.2. Ferric Reducing/Antioxidant Power (FRAP) Assay

The reducing ability of the Fe^3+^ ion to Fe^2+^ ion under acidic conditions was evaluated using the FRAP assay. This antioxidant activity was evaluated by monitoring the formation of a Fe^2+^–TPTZ complex with a spectrophotometer (Jasco Inc., Easton, MD, USA). The FRAP working solution was prepared by mixing 10 vol of 0.3 M acetate buffer, pH 3.6 (3.1 g sodium acetate and 16 mL glacial acetic acid), 1 vol of 10 mM TPTZ prepared in 40 mM HCl, and 1 vol of 20 mM FeCl_3_. All the components of the working solutions were freshly prepared and used on the same day of preparation. Before performing the assay, all the solutions were brought to 37 °C. The amount of 2.85 mL of working solution was mixed with 0.15 mL diluted samples and incubated at 37 °C for 4 min. After the incubation time, the absorbance was acquired at 593 nm (Jasco Inc., Easton, MD, USA). The blank was represented by the only working solution. For the calculation of antioxidant activity, the blank absorbance value was subtracted from the absorbances of the samples. All analyses were performed in triplicate. A standard curve was plotted with Trolox, and the results are expressed as µmol TE.

#### 2.7.3. ABTS^•^ Radical Scavenging Assay

The ABTS assay is an antioxidant protocol based on the ability of the molecules to react with ABTS^•+^ radicals (2, 20-azinobis (3-ethylbenzotiazoline-6-sulfonate)). The test was performed according to the experimental protocol previously performed by Maisto et al. (2022) [31], with some modifications. ABTS solution was obtained by shaking 2.5 mL of an ethanolic solution of ABTS 7.0 mM with 44 µL of an aqueous solution of potassium persulfate 140 mM, which was stored for at least 7 h at 5 °C in darkness. After this time, the working solution was prepared by diluting the obtained mixture with an ethanol-water solution until an absorbance value of 0.700 ± 0.05 was acquired at 754 nm (Jasco Inc., Easton, MD, USA). The assay was performed by mixing 1000 µL ABTS working solution with 100 µL of the sample previously diluted in the extraction solvent. The mixture was incubated for 2.5 min in the darkness. After this time, the sample absorbances were read at 734 nm. The control was obtained by replacing the samples with the same volume of ethanol. The radical inhibition was calculated according to the formula: [(A_i_ − A_f_ )/A_c_] × 100, where A_i_ is the absorbance of the sample at t = 0, A_f_ is the absorbance after 2.5 min, and A_c_ is the absorbance of the control at time zero. The antioxidant standard used as a positive control was Trolox. The results are expressed both as µmol of TE and EC_50_, which is the amount of antioxidant necessary to decrease the initial ABTS^•+^ concentration by 50%.

### 2.8. Skin Sample

Four mm skin flaps of pig (age of 8–9 years) ears were excised from the outer part of a male pig ear, post-sacrifice, within 24 h from animal death, and left to settle at a controlled temperature and humidity of 24 °C and r.H. of 50.0% for approximately 30 min. Dial Calipers-0–4-Inch-001 Inch (52-008-704 SU, WESTport, Corporation, West Islip, New York 11795) was used to ensure skin thickness, and Tewameter TM Nano (C + K electronic GmbH, Köln, Germany) was used to ensure skin integrity, accepted TEWL values were <15 g/mh^2^. Then, skin samples were loaded into the diffusion cell system.

#### 2.8.1. Diffusion Experiment

Franz’s diffusion cell system consists of 2 main chambers: the donor and the receptor, between which the excised porcine skin flap, stripped of its subcutaneous tissue, is placed, with the epidermis facing the donor compartment and fixed with forceps. Triplicate Franz cells were fitted for each oleolyte to be analyzed, plus 1 for the blank. The donor compartment of each cell was loaded with 1 mL of oleolyte containing 211 µg/mL of UA. While the receptor was filled with 5 mL of PBS solution and a magnet to avoid the saturation of the first liquid layer in contact with the skin. After assembly, the cells are placed in a thermostatically controlled bath at 37.0 °C. A special cap is mounted on the donor compartment to maintain a constant pressure (1 atm) inside the chamber. A total of 24 Franz cells were prepared, 12 with the oleolyte and 12 for blank. After placement, the time is monitored with a timer, and 1 mL of the receptor fluid is collected at set times. Finally, cells are disassembled, and the skin is collected for further extraction procedures. Skin flaps were rinsed with physiological saline solution (NaCl 0.9%) by dabbing with a cotton pad (Linea F, Angelini Acraf Spa, Aprilia, Italy), which was then cut into a 10 mm diameter piece. Before extractions, the epidermis and dermis were split by heating with a hair dryer for 30 secs, then by scraping with tweezers (Semken-Taylor Rette N. 1 Cm. 12, 5, Asa Dental Spa) and surgery (Pikdare Spa, Casnate con Bernate, Como, Italy) [32,33]. Afterward, the epidermis and dermis were placed in individual glass test tubes and kept in contact with 1 mL of an ethanolic solution containing PBS for 1 h. Subsequently, the solvent was gently evaporated under N_2_ flow. The experiments were carried out employing 2 different concentrations, the higher of 50 μg/mL, out of iBuP, which was 100 μg/mL, and the lower of 5 μg/mL, out of iBuP, which was 10 μg/mL, respectively. Extraction from each skin layer (Epidermis and Dermis) was carried out using 1 mL of water: ethanol sol. 50/50 (*v*/*v*) as an extraction solvent. The mixtures were sonicated for 30 min at 30 °C and finally left in agitation (600 rpm) at room temperature for 4 h. After the extraction time, skin samples were discharged, and the extraction solvent was transferred to plastic vials and then centrifuged at 12,000 rpm for 10 min. The supernatant was filtered using nylon syringe filters 0.22 µm (Phenomenex, Bologna, Italy) and analyzed by HPLC-MS.

#### 2.8.2. HPLC-MS Method for Franz’s Diffusion Cell System

An HPLC Agilent 1200 coupled with an Agilent Technologies 6470-triple quadrupole mass spectrometer (Agilent Technologies, Palo Alto, CA, USA) was used for the analysis. Elution was performed on a Kinetex^®^ XB-C18 column (50 mm × 3 mm, 2.6 μm; Phenomenex, Torrance, CA, USA). The mobile phases were water with 0.1% formic acid (A) and methanol with 0.1% formic acid (B). The elution gradient was performed according to the following conditions: 0–3 min, isocratic on 40% phase B; 3–13 min, linear gradient from 40 to 95% B; 13–18 min, isocratic on 95% B; 18–21 min, isocratic on 40% B for column recondition. The column temperature was set at 40 °C, inject volume was 5 µL, and the flow rate was set at 0.40 mL/min. The source was a heated electrospray interface (HESI) operated in positive ionization with multiple reaction monitoring (MRM) scanning modes. One MRM transition was used as a quantifier transition and a second MRM transition served as a qualifier transition. The first transition (455.3–455.3) was made with a collision energy of 20 eV, while the second transition (455.3–407.0) was performed with a collision energy of 40 eV. The 2 transitions were made with a fragmentor of 135. Argon was used as a gas for collision-induced fragmentation. The MRM analyte parameters were optimized using a methanolic solution of ursolic acid at a concentration of 1 ppm. Ursolic acid was quantified according to a calibration curve (R^2^ ≥ 0.99) made with 9 different concentrations (5000, 1000, 500, 100, 50, 10, 5, 1, and 0.5 ppb) and triplicate injections at each concentration. The ion source was set using the following parameters: gas temperature: 270 °C; gas flow rate: 10 L/min; gas temperature: 300 °C; sheath gas flow rate: 12 L/min; capillary voltage: 3500 V; nebulizer pressure: 40 psi; and nozzle voltage: 1000 V.

### 2.9. Statistics

Unless otherwise stated, all the experimental results were expressed as the mean ± standard deviation (SD) of 3 repetitions. Graphics and IC_50_ values determination were calculated using GraphPad Prism 8 software. The RSM optimization was performed with Minitab software version 21.1.0. The RSM optimization was performed by applying the variance ANOVA analysis and the Pareto-chart graphic to identify the significant process parameters. These parameters were used in a multiple-response prediction analysis to identify a polynomial model to optimize UA concentration.

## 3. Results and Discussion

### 3.1. Optimisation of Ursolic Acid Extraction Using RSM Model

The choice to optimize the UA extraction conditions in sunflower oil was related to its ability to extract mainly the AA lipophilic components such as UA. In the literature, mainly ethyl acetate, chloroform, and hexane have been described as exhaustive solvents for UA recovery from natural sources [1,34]. Since these solvents are not considered biocompatible, the obtained extract cannot be suitable for the preparation of formulations for human usage. Therefore, there is an urgent need to individuate an apolar biocompatible solvent for the recovery of lyophilic natural compounds from the food matrix. To this end, we have optimized the extraction of UA from AA in sunflower oil using the RSM statistical model.

**Figure 1:**
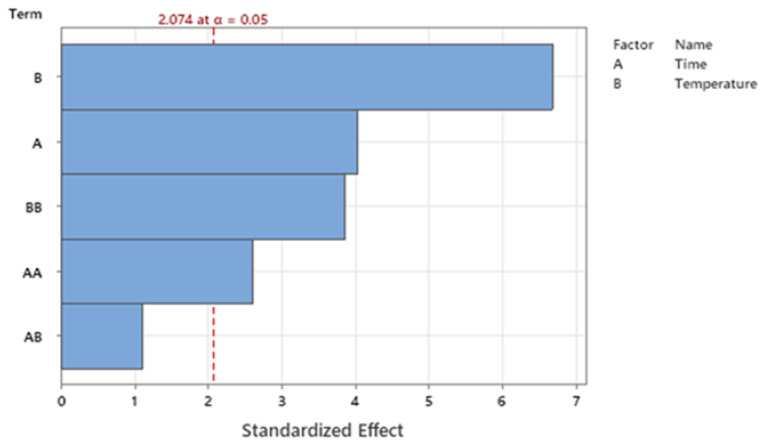
Pareto chart of significative and not significative parameters analyzed, i.e., extraction time (A) and temperature (B).

According to our experimental protocol, the extraction temperature was constantly kept below 80 °C, considering that other works have reported that the UA extraction rate decreases above 70 °C [35]. Our experimental data showed that UA concentration ranged from 8.21 ± 0.41 µg/mL (*p* < 0.001; 60 min, 20 °C, 1 h, with 30 min of sonication) to 734.79 µg/mL (68.85 min, at 63 °C, without sonication). First, three independent commonly modified factors, i.e., extraction time (12, 24, 48, and 96 h) and temperature (20, 40, 60, and 80 °C), combined with or without a single sonication cycle (30 min), were evaluated to optimize the UA yield in sunflower oil. Considering the independent variables analyzed, according to the preliminary ANOVA analysis, only extraction time (A) and extraction temperature (B), without sonication treatment (C), were significantly correlated with UA extraction yield, as described in the Pareto-chart graphic, with α = 0.05 (Figure 1).

According to the statistical results of model fitting, the best model to maximize the UA yield would be achieved by limiting the statistical analysis to a two-variable correlation (2 FI, i.e., A and B) (Figure 1). The multiple regression analysis of UA amounts demonstrated that the model was significant (*p* < 0.0001), did not present a lack of fit *(p* = 0.182), and the predictivity of the model was 77.89% (R-sq 84.47%, R-sq (adj) 80.95%; R-sq (pre) 77.89%). Second-order quadratic polynomial models were suitable to assess the influence of the two independent and significative variables on the UA output, as described by Equation (1), in terms of uncoded units, where variables A and B were extraction time and temperature, respectively.

UA Concentration (µg/mL) = 11.07 A + 30.55 B − 0.0685A^2^ − 0.2782B^2^ − 0.0336 AB − 624 (1)

Equation (1) suggests that UA extraction in oil is both a time- and temperature-dependent process. Specifically, according to Equation (1), factors A and B influence in different manners UA output. Precisely, considering that the B factor is associated with a high numeric coefficient (30.55 and 0.278), extraction temperature has a more pivotal role in influencing UA yield than extraction time (A).

Generally, according to other works related to the optimization of the UA extraction process in ethanol from food sources, the optimum temperature values ranged from 40 to 50 °C [36,37]. Our model seems to indicate that the most effective temperature is 63 °C. This difference, in comparison to the findings above reported, probably may be related to the higher viscosity of sunflower oil used as a solvent, in comparison to the organic solvents conventionally employed for UA recovery. It was well established that high extraction temperatures decrease the viscosity of both the extraction medium and the solvent (sunflower oil), which helps the solvent (more fluid) penetrate the plant matrix, resulting in faster kinetics and exhaustive solvents [38]. However, the increase in solvent temperature may reduce surface tension and, consequently, improve the permeability of the food matrix, resulting in a higher extraction rate [39].

**Figure 2:**
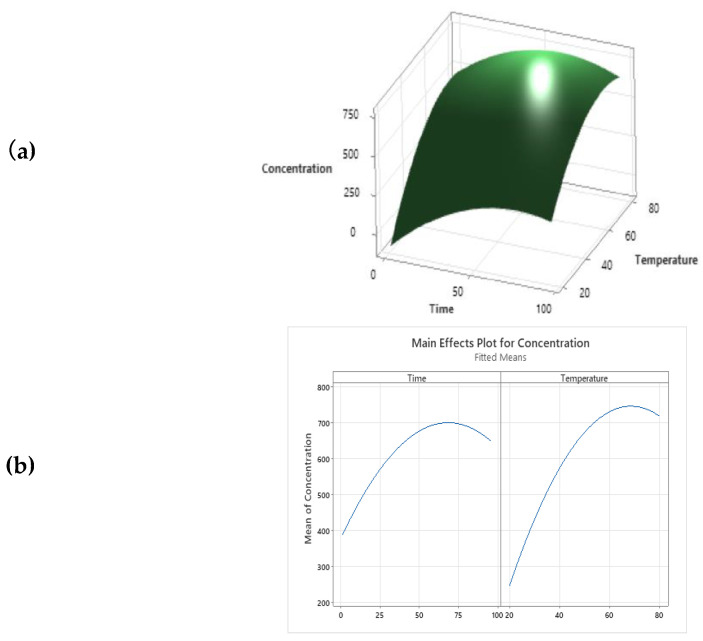
(**a**) Multiple response prediction analysis; (**b**) Surface plot of UA concentration (µg/mL) *cv* time (h) and temperature (°C).

Furthermore, as reported by Equation (1), the temperature value must be kept below maximum values, which causes the degradation of UA [35]. The same results were reported by the 3D response surface graphic, which correlates all the variables investigated (Figure 2a). The predictive model estimates the theoretical conditions to obtain the optimal UA extraction in oil solvent at 63 °C for 68.85 h, as reported in Figure 2. These two parameters were combined to set up new extraction conditions from AA to verify and confirm the theoretical UA yield of 761.50 µg/mL, as shown by the multiple response prediction analysis performed.

Our experimental data reported that UA concentration obtained in optimized condition was 784.40 ± 7.579 mg/mL (*p* < 0.0001), with an EA% of 3%. This evidence confirms the predictivity of the developed extraction method [40].

### 3.2. Quantitative Polyphenolic Analysis of OAAO by HPLC-DAD-FLD

In order to establish the polyphenolic composition of OAAO obtained, the chromatographic analysis of the hydroalcoholic OAAO extract was performed using a method previously optimized and developed [27]. The HPLC-DAD-FLD analysis led to the identification and quantification of 7 phenolic compounds. The observed data are listed in Table 2. Predictably, considering the great structural diversity of the AA polyphenolic composition, which ranged from polar molecules (e.g., phenolic acids) to lipophilic compounds (e.g., de-glycosylated flavonols), only the AA polyphenolic lipophilic fraction was identified in OAAO. Specifically, rutin and quercetin-3-*O*-glucoside are the most abundant polyphenols occurring in OAAO. Apart from flavonols, dihydrochalcones are the second most representative polyphenolic class in OAAO, with phlorizin and phloretin reaching concentrations of 0.15 µg/mL and 0.07 µg/mL, respectively. As reported in Table 2, the more hydrophilic AA polyphenolic compounds were not detected in OAAO. This is related to the high lipophilicity of sunflower oil used as a solvent, which was able to selectively extract only the non-polar polyphenolic AA compounds. Quercetin, on the other hand, was not detected in OAAO, despite its high lipophilicity. This result may be related to the low initial quercetin concentration in AA, which may reach an undetectable concentration in OAAO.

### 3.3. Qualitative Polyphenols and Terpenoid Characterisation by HPLC-HESI-MS/MS

OAAO was subjected to a double and different extraction procedure, in both hydroalcoholic and ethyl acetate solvents, as previously described in Section 2.3.1 and Section 2.4.1, in order to evaluate its chemical composition. The hydroalcoholic extract was analyzed to assess the qualitative OAAO polyphenolic composition, while OAAO ethyl acetate extract was analyzed to evaluate its terpenoid composition, anyway, all the identified compounds were reported in Table 3. Quercetin *O*-rutinoside (compound **1**) showed a [M-H]^−^ ion at *m*/*z* 609. The base peak ion at 301 [M-H-Rut]^−^, derived from the cleavage of the disaccharide group, and the fragment ions at *m*/*z* 255 [M-H-Rut-CO-H_2_O]^−^ and at *m*/*z* 179 [M-H-Rut-C_7_H_6_O_2_]^−^, which derived from the RDA fragmentation, agreed with literature dat [41]. However, compound **1** was identified as rutin by comparison with an authentic analytical standard. Compound **2** displayed a [M-H]^−^ ion at *m*/*z* 463. The base peak ion at *m*/*z* 301 [M-H-Glu]^−^ was derived from the neutral loss of glucose moiety while the fragment ion at *m*/*z* 179 [M-H-Glu-C_7_H_6_O_2_]^−^ was due to the RDA fragmentation. Based on the tandem mass spectrum and by comparison with an analytical standard, compound **2** was identified as quercetin 3-*O*-glucoside [41]. Compound **3** displayed a [M-H]^−^ ion at *m*/*z* 447 and was annotated as kaempferol 3-*O*-glucoside. Its tandem mass spectrum showed a base peak ion at *m*/*z* 285 [M-H-Glu]^−^, due to the loss of the glucose group, and the fragment ions at *m*/*z* 179 [M-H-Glu-C_7_H_6_O_2_]^−^, due to the RDA fragmentation, confirmed the linkage of the glucose moiety at the aglycone kaempferol. Based on literature data and by comparison with an authentic analytical standard, compound **3** was identified as kaempferol 3-*O*-glucoside [42]. Compound **4** showed a [M-H]^−^ ion at *m*/*z* 435. The base peak ion at *m*/*z* 273 [M-H-Glu]^−^ for the loss of the glucose unit and the fragment ion at *m*/*z* 167 [M-H-Glu-C_7_H_6_O]^−^ confirmed the presence of the chalcone moiety and the linkage with a hexoside group. Based on the tandem mass spectrum and by comparison with an authentic analytical standard, compound **4** was identified as phloridzin [41]. Kaempferol 3-*O*-rhamnoside (compound **5**) displayed a [M-H]^−^ ion at *m*/*z* 431. The base peak ion at *m*/*z* 285 [M-H-Rha]^−^ was derived from the neutral loss of the sugar moiety while the fragment ion at *m*/*z* 179 [M-H-Rha-C_7_H_6_O]^−^ was due to the RDA fragmentation [43]. Compound **5** identity was confirmed by comparison with an authentic analytical standard. Compound **6** displayed a [M+H]^+^ ion at *m*/*z* 503. The base peak ion at *m*/*z* 485 [M+H-H_2_O]^+^ and the fragment ions at *m*/*z* 467 [M+H-2H_2_O]^+^, at *m*/*z* 457 [M+H-HCOOH]^+^ and at *m*/*z* 439 [M+H-HCOOH-H_2_O]^+^ indicated the linkage of a hydroxyl and a carboxylic acid groups. Based on literature data, compound **6** was putatively identified as hydroxymethoxyursolic acid [44]. Compound **7** showed a [M-H]^−^ ion at *m*/*z* 273. The base peak ion at *m*/*z* 167 [M-H-C_7_H_6_O]^−^ and the fragment ion at *m*/*z* 125 [M-H-C_9_H_8_O_2_]^−^ indicated the presence of the chalcone moiety. Based on the tandem mass spectrum and by comparison with authentic analytical standard, compound **7** was identified as phloretin. Compound **8** displayed a [M+H]^+^ ion at 501. The base peak ion at *m*/*z* 455 [M+H-HCOOH]^+^ and the fragment ion at *m*/*z* 419 [M+H-HCOOH-2H_2_O]^+^. By comparison with literature data, compound **8** was putatively annotated as carboxyursolic acid [41]. Compound **9** showed a [M+H]^+^ ion at *m*/*z* 487 and was tentatively identified as annurcoic acid [22], a peculiar triterpenoid acid isolated exclusively in Annurca Apple. The tandem mass spectrum displayed a base peak ion at *m*/*z* 469 [M+H-H_2_O]^+^ and a fragment ion at *m*/*z* 423 [M+H-HCOOH-H_2_O]^+^, suggested the linkage of carboxylic and hydroxy groups. Compound **10** displayed a [M-H]^−^ ion at *m*/*z* 517. The base peak ion at *m*/*z* 455 [M-H-CO_2_-H_2_O]^−^ and the fragment ion at *m*/*z* 429 [M-H-2CO_2_]^−^ suggested the linkage of two carboxylic groups and a hydroxy moiety. Its tandem mass spectrum allowed to putatively identify the compound **10** as zahnic acid [45]. Two medicagenic acid isomers (compounds **11** and **16**) were tentatively detected. They showed a [M-H]^−^ ion at *m*/*z* 501 and a base peak ion at *m*/*z* 483 due to the neutral loss of a molecule of water. The fragment at *m*/*z* 391 [M-H-2HCOOH-H_2_O]^−^ indicated the presence of two carboxylic groups and agreed with the literature data [44]. Compound **12** showed a [M+H]^+^ ion at *m*/*z* 473 and was putatively identified as corosolic acid. The base peak ion at *m*/*z* 455 [M+H-H_2_O]^+^ and the fragment ion at *m*/*z* 391 [M+H-HCOOH-2H_2_O]^+^ were due to the neutral losses of water and formic acid molecules [46]. Compound **13** displayed a [M-H]^−^ ion at *m*/*z* 487. Its tandem mass spectrum was characterized by a base peak ion at *m*/*z* 425 [M-H-CO_2_-H_2_O]^−^ and some fragment ions at *m*/*z* 441 [M-H-HCOOH]^−^ and at *m*/*z* 407 [M-H-CO_2_-2H_2_O]^−^, which indicated the linkage of a hydroxy group and a carboxylic acid. Based on literature data, compound **13** was putatively identified as arjunolic acid [46]. Compound **14** showed a [M+H]^+^ ion at *m*/*z* 457 and was tentatively annotated as oleanolic acid. The base peak ion at *m*/*z* 439 [M+H-H_2_O]^+^, due to the neutral loss of a molecule of water, and the fragment ions at *m*/*z* 393 [M+H-HCOOH-H_2_O]^+^, derived from the cleavage of the hydroxy and the carboxy groups, confirmed oleanolic acid mass spectrum and agreed with literature data [46]. Compound **15** displayed a [M+H]^+^ ion at *m*/*z* 457. The base peak ion at *m*/*z* 411 [M+H-HCOOH]^+^ and the fragment ion at *m*/*z* 393 [M+H-HCOOH-H_2_O]^+^ indicated the presence of a carboxylic acid and a hydroxy group. Based on the tandem mass spectrum and by comparison with an authentic analytical standard, compound **15** was identified as ursolic acid [46]. Compound **17** showed a [M-H]^−^ ion at *m*/*z* 633. The base peak ion at *m*/*z* 589 [M-H-CO_2_]^−^ and the fragment ion at *m*/*z* 571 [M-H-CO_2_-H_2_O]^−^ derived from the cleavage of the carboxy the hydroxy groups. However, the fragment ion at *m*/*z* 487 [M-H-CA]^−^ indicated the linkage of the coumaric acid moiety. Based on literature data, compound **17** was putatively annotated as dihydroxy-{[(hydroxyphenyl)-propenoyl)]oxy}ursenoic acid [47]. Compound **18** displayed a [M-H]^−^ ion at *m*/*z* 617. The base peak ion at *m*/*z* 573 [M-H-CO_2_]^−^ and the fragment ion and the fragment ion at *m*/*z* 453 [M-H-CA-H_2_O]^−^ suggested the linkage of the carboxylic acid, the hydroxyl, and the coumaric acid groups. Based on literature data, compound **18** was tentatively identified as hydroxy-{[(hydroxyphenyl)-propenoyl)]oxy}ursenoic acid [47].

### 3.4. Validation of Ursolic Acid HPLC-DAD Analysis Method

The HPLC-DAD method for the characterization and quantification of UA in OAAO was validated. To this purpose, the linearity, sensitivity, accuracy, and precision of the method were evaluated. Specifically, linearity studies were conducted by generating calibration curves on a wide range of standard analytical dilutions (six concentrations ranging from 0.001 to 0.5 mg/mL). All analyses were performed in triplicate, and standard concentrations were plotted versus peak area, yielding a linear relation (Table 4), described by a correlation coefficient of R^2^ 0.999. The sensitivity of the analytical method was assessed by determining the UA LOD e LOQ values. Our results show that UA LOD is 0.295 ppm, while UA LOQ is 0.845 ppm (Table 4). These values are perfectly in line with the results of other authors, who reported values of UA LOD and LOQ of 0.15 ppm and 0.47, respectively [47]. Since UA LOD and LOQ values are largely below the concentrations detected and quantified in all samples analyzed, this analytical method can be considered a reliable protocol for both UA detection and quantification.

Intra-day and inter-day accuracy (% bias), and precision (% C.V.), were determined at UA concentrations of 500, 100, and 5 ppm (Table 5). As expected, the higher % C.V. was measured at, the lower concentration tested (5 ppm), with intra-day and inter-day % C.V. of 5.773% and 7.375%, respectively. The same was for the accuracy, whose lower values of % bias were obtained at the lower concentration tested, with a % bias of −0.534% (intra-day) and of −0.521% (inter-day). In general, we found that % C.V. values ranged from 1.874 to 5.773% and from 1.720 to 7.375% for intra-day and inter-day precision, respectively. Moreover, the % bias ranged from −0.534 to 3.142% for the estimation of intra-day accuracy and from −1.386 to −0.521% for the evaluation of inter-day accuracy. Considering low values obtained for both %bias and %CV, the developed method may be considered a reproducible and reliable protocol for the quantification of UA by HPLC-DAD analysis.

The recovery (%) and the matrix effect (%) were determined at UA concentrations of 30, 20, and 10 µg (Table 6). As expected, the higher % matrix effect was measured at the lower Ursolic acid spiked concentration (10 µg), with a % matrix effect of 6.86%. In general, we found that % recovery values and % matrix effect ranged from 89.42 to 91.21% and from 1.42 to 6.86%, respectively. Considering low values obtained for both % recovery values and % matrix effect, the developed extraction and analysis methods may be considered as a reproducible and reliable protocol for the quantification of UA by HPLC-DAD analysis.

### 3.5. Total Polyphenols and In Vitro Antiradical Activity of OAAO

According to current knowledge, oxidative stress and UV irradiation are the main causes of extrinsic and premature aging, as well as some skin cutaneous damage and diseases [48]. Oxidative stress plays a pivotal role in the skin aging process and in several age-related chronic diseases. Reducing ROS has been shown to attenuate oxidative damage and extend animal tissues’ lifespan [49]. Considering the potential incorporation of OAAO in cosmetic formulations, its total phenolic content (TPC) and in vitro antiradical activity were investigated. To evaluate the OAAO total polyphenolic content, the Folin–Ciocalteau’s assay was performed on OAAO hydroalcoholic fraction, resulting in 5.56 ± 0.45 GAE (gallic acid equivalent)/g of OAAO. On the other hand, the antiradical activities of OAAO were tested by the application of DPPH, ABTS and FRAP protocol, and the obtained results are shown in Table 7.

**Figure 3:**
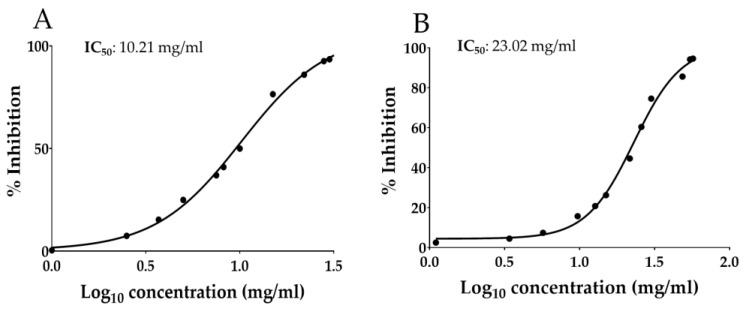
Antiradical activity of Optimized Annurca Apple Oleolyte (OAAO) hydroalcoholic extract expressed as (**A**) IC_50_ of DPPH assay and (**B**) IC_50_ of ABTS assay. Values represent the mean ± standard deviation of triplicate readings.

The scientific literature reported that the antiradical activity of Annurca apple polyphenols was 40.98 ± 1.19 for ABTS, 15.59 ± 0.11 for DPPH, and 26.63 ± 0.70 µmol TE/g DW for FRAP assay [50]. Interestingly, although the extraction in oil is highly selective for the lipophilic molecules, OAAO has shown valuable antiradical potential (Table 7). These results could be related to the non-polar apple polyphenols, such as flavonols and dihydrochalcones, which are the main polyphenolic components in OAAO, as previously described in Section 3.2 and Section 3.3. Furthermore, the results of the DPPH and ABTS assays were also estimated as IC_50_ (Figure 3), which is defined as the amount of antioxidants required to reduce the initial concentration of the radical solution by 50% [51]. Regarding the reported OAAO IC_50_ values, also in this case, the antiradical activity registered by the DPPH assay (10.21 mg/mL) was higher (doubled) than those acquired by the ABTS assay (23.02 mg/mL). The reported trend is the same previously described by the estimation of the antiradical activity expressed as µmol TE/g DW (14.63 ± 0.22 and 5.90 ± 0.49, respectively, calculated by DPPH and ABTS assays).

### 3.6. Skin Permeation Study

In order to evaluate the potential application of OAAO for the formulation of natural cosmetic products, a study of skin penetration was performed. The passage of natural compounds across the major two skin layers (epidermis and derma) is a complex process involving several stages: (i) the release of the molecule from the vehicle, (ii) penetration into the stratum corneum, and (iii) partitioning from stratum corneum to target sites in epidermis and dermis [52]. Our results indicate that the oleolyte containing UA has a promising penetration rate after 1, 2 and 4 h of application and reaches its maximum concentration in the derma compartment after 4 h after application. No edema and erythema were observed after the administration of the oleolyte to porcine skin.

**Figure 4:**
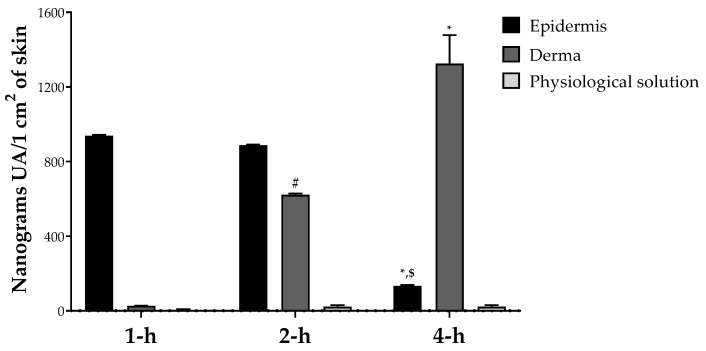
UA in vitro skin permeation study; values are presented as means ± Dev. st of three replicates. Data were analyzed with two-way ANOVA followed by Tukey’s posthoc test; * *p* < 0.01 4-h vs. 1-h, # *p* < 0.01 2-h vs. 1-h, $ *p* < 0.01 4-h vs. 2-h in the same group.

The LC-MS/MS measurements showed that the UA distribution was linear over time, with a progressive shift from the epidermal to the dermal compartment, but without ever affecting the blood flow simulated by the physiological solution (Figure 4). These valuable results may be related to the triterpenoid lipophilic structure of UA, characterized by XLogP3-AA of 7.3. In general, the topically applied compound can penetrate the skin by two different routes, transcellular and intercellular, depending on the structural features of the compound under study. Considering that lipophilic molecules prefer intercellular passage [53], UA can potentially diffuse across the different skin compartments via the intercellular route. The Regulation (EC) No. 1223/2009 of the European Union defines a cosmetic product as “any substance or mixture intended to come into contact with the external parts of the human body (epidermis, hair system, nails, lips, and external genital organs) or with the teeth and mucous membranes of the oral cavity with a view exclusively or mainly to cleaning them, perfuming them, changing their appearance, protecting them, keeping them in good condition or correcting body odors” [33]. Consequently, the main characteristic that distinguishes cosmetics from pharmaceutical products is that the cosmetic product must not enter the bloodstream [33]. As reported in our permeation study, only a statistically not significant trace of UA was observed in the physiological region (which simulates the blood circulation in our system) (Figure 4). Based on these data, OAAO can be considered a powerful and safe functional ingredient for the formulation of a cosmetic product.

## 4. Conclusions

The described results show that AA could be considered a possible source of bioactive compounds, especially UA. Notably, optimization of the extraction conditions using the RSM methodology allowed us to evaluate the maximum extractable UA amount in AA (784.40 ± 7.579 µg/mL) using sunflower seed oil as a food-grade extraction solvent. The extract obtained in the optimized conditions (OAAO) was also characterized to assess its chemical composition and its in vitro potential biological activities. The promising results in terms of antioxidant properties (IC_50_ 10.21 mg/mL and 23.02 mg/mL evaluated respectively by DPPH and ABTS assays) and skin permeation (reaching maximum UA concentration in the epidermis after 2 h of treatment) may suggest OAAO as a powerful functional ingredient for the formulation of cosmetic products with anti-aging effects. Further investigations on the in vitro and in vivo beneficial potential, especially the potential blanching activity, are necessary.

## Figures and Tables

**Table 1:** Independent variables used for the RSM model set.

Independent Variables	Factor Levels						
Time (hours)	1	2	4	12	24	48	96
Temperature (°C)	20	40	60	80			
Total runs	28						

**Table 2:** Quantitative analysis of OAAO evaluated by HPLC-DAD-FLD analysis.

Compound	Mean Value ± SD (µg/mL of OAAO)
Chlorogenic acid	nd
Caffeic acid	nd
*p*-Cumaric acid	nd
Procyanidin B1 + B3	nd
Procyanidin B2	nd
Epicatechin	nd
Rutin	0.76 ± 0.001
Quercetin-3-*O*-glucoside	0.71 ± 0.007
Kaempferol-3-*O*-rhamnoside	0.15 ± 0.010
Kaempferol-3-*O*-glucoside	LOQ
Apigenin-7-*O*-glucoside	0.0081 ± 0.0001
Phloridzin	0.15 ± 0.010
Quercetin	nd
Phloretin	0.07 ± 0.01

Values are expressed in µg/mL of OAAO ± standard deviation (SD) of three repetitions. nd: not detected.

**Table 3:** HPLC-HESI–MS/MS analysis of OAAO hydroalcoholic and ethyl acetate extracts.

No.	Extract	Compound	Ione	Rt	*m*/*z*	Diagnostic Fragment	Ref.
1	Hydroalcoholic	Rutin	[M-H]^−^	8.05	609	591 [M-H-H_2_O]^−^, 301 [M-H-Rut]^−^, 255 [M-H-Rut-CO-H_2_O]^−^, 179 [M-H-Rut-C_7_H_6_O_2_]^−^	[41]
2	Hydroalcoholic	Quercetin-3-*O*-glucoside	[M-H]^−^	8.16	463	445 [M-H-H_2_O]^−^, 301 [M-H-Glu]^−^, 255 [M-H-Glu-CO-H_2_O]^−^, 179 [M-H-Glu-C_7_H_6_O_2_]^−^	[4]
3	Hydroalcoholic	Kaempferol-3-*O*-glucoside	[M-H]^−^	8.73	447	429 [M-H-H_2_O]^−^, 285 [M-H-Glu]^−^, 179 [M-H-Glu-C_7_H_6_O_2_]^−^, 151 [M-H-Glu-C_8_H_6_O_3_]^−^	[41]
4	Hydroalcoholic	Phloridzin	[M-H]^−^	9.41	435	417 [M-H-H_2_O]^−^, 273 [M-H-Glu]^−^, 167 [M-H-Glu-C_13_H_16_O_6_]^−^	[40]
5	Hydroalcoholic	Kaempferol-3-*O*-rhamnoside	[M-H]^−^	9.78	431	413 [M-H-H_2_O]^−^, 327 [M-H-C_4_H_8_O_3_]^−^, 285 [M-H-Rha]^−^, 179 [M-H-Rha-C_7_H_6_O_2_]^−^	[42]
6	Ethylacetate	Hydroxymethoxyursolic acid	[M+H]^+^	11.49	503	485 [M+H-H_2_O]^+^, 467 [M+H-2H_2_O]^−^, 457 [M+H-HCO_2_H]^+^, 439 [M+H-HCO_2_H-H_2_O]^+^	[43]
7	Hydroalcoholic	Phloretin	[M-H]^−^	11.61	273	255 [M-H-H_2_O]^−^, 167 [M-H-C_7_H_6_O]^−^, 125 [M-H-C_9_H_8_O_2_]^−^	[40]
8	Ethylacetate	Carboxyursolic acid	[M+H]^+^	12.17	501	483 [M+H-H_2_O]^+^, 455 [M+H-HCO_2_H]^+^, 437 [M+H-HCO_2_H-H_2_O]^+^, 419 [M+H-HCO_2_H-2H_2_O]^+^	[43]
9	Ethylacetate	Annurcoic acid	[M+H]^+^	13.47	487	469 [M+H-H_2_O]^+^, 451 [M+H-2H_2_O]^−^, 441 [M+H-HCO_2_H]^+^, 423 [M+H-HCO_2_H-H_2_O]^+^	[20]
10	Ethylacetate	Zanhic acid	[M-H]^−^	14.83	517	499 [M-H-H_2_O]^−^, 473 [M-H-CO_2_]^−^, 455 [M-H-CO_2_-H_2_O]^−^, 429 [M-H-2CO_2_]^−^	[44]
11	Ethylacetate	Medicagenic acid isomer 1	[M-H]^−^	15.09	501	483 [M-H-H_2_O]^−^, 457 [M-H-CO_2_]^−^, 409 [M-H-2HCOOH]^−^, 391 [M-H-2HCOOH-H_2_O]^−^	[44]
12	Ethylacetate	Corosolic acid	[M+H]^+^	15.19	473	455 [M+H-H_2_O]^+^, 427 [M+H-HCO_2_H]^+^, 409 [M+H-HCO_2_H-H_2_O]^+^, 391 [M+H-HCO_2_H-2H_2_O]^+^	[45]
13	Ethylacetate	Arjunolic acid	[M-H]^−^	15.75	487	469 [M-H-H_2_O]^−^, 441 [M-H-HCOOH]^−^, 425 [M-H-CO_2_-H_2_O]^−^, 407 [M-H-CO_2_-2H_2_O]^−^	[45]
14	Ethylacetate	Oleanolic acid	[M+H]^+^	15.91	457	439 [M+H-H_2_O]^+^, 411 [M+H-HCO_2_H]^+^, 393 [M+H-HCO_2_H-H_2_O]^+^	[45]
15	Ethylacetate	Ursolic acid	[M+H]^+^	16.20	457	439 [M+H-H_2_O]^+^, 411 [M+H-HCO_2_H]^+^, 393 [M+H-HCO_2_H-H_2_O]^+^	[45]
16	Ethylacetate	Medicagenic acid isomer 2	[M-H]^−^	16.67	501	483 [M-H-H_2_O]^−^, 457 [M-H-CO_2_]^−^, 439 [M-H-CO_2_-H_2_O]^−^, 391 [M-H-2HCOOH-H_2_O]^−^	[44]
17	Ethylacetate	Dihydroxy-{[(hydroxyphenyl)-propenoyl)]oxy}ursenoic acid	[M-H]^−^	18.79	633	615 [M-H-H_2_O]^−^, 589 [M-H-CO_2_]^−^, 571 [M-H-CO_2_-H_2_O]^−^, 487 [M-H-CA]^−^	[46]
18	Ethylacetate	Hydroxy-{[(hydroxyphenyl)-propenoyl)]oxy}ursenoic acid	[M-H]^−^	20.89	617	599 [M-H-H_2_O]^−^, 573 [M-H-CO_2_]^−^, 453 [M-H-CA-H_2_O]^−^	[46]

**Table 4:** Linearity and sensitivity of the HPLC-DAD method.

Compound	Linearity	Correlation Coefficient (*r*^2^)	LOQ (ppm)	LOD (ppm)	Monitoring Channel
Ursolic acid	Y = 4 E + 06 x + 32,787	0.999	0.845	0.295	205 nm

**Table 5:** Intra-day and inter-day precision and accuracy of UA.

Analyte Concentration (ppm)		Intra-Day (CV%, *n* = 3)	Inter-Day (CV%, *n* = 3)	Intra-Day (%Bias, *n* = 3)	Inter-Day (%Bias, *n* = 3)
	500	1.874	1.720	3.142	1.386
Ursolic acid	100	1.960	0.885	0.580	1.120
	5	5.773	7.375	−0.534	−0.521

**Table 6:** Recovery and matrix effect of UA.

Analyte Ursolic Acid Spiked (µg)		Recovery (%)	Matrix Effect (%)
	30	89.65	2.29
Ursolic acid	20	89.42	1.42
	10	91.21	6.86

**Table 7:** Antiradical activity of OAAO extracts evaluated by DPPH, ABTS and FRAP assays.

Antioxidant Activity (µmol TE/g of OAAO Hydroalcoholic Extract ± SD)		
DPPH Assay	ABTS Assay	FRAP Assay
14.63 ± 0.22	5.90 ± 0.49	21.72 ± 0.68

The results are expressed as µmol TE per gram of AA. Abbreviations: DPPH, (2, 2diphenyl-1-picrylhydrazyl; ABTS, 2, 20-azino-bis (3-ethylbenzothiazoline-6-sulfonic acid); FRAP, ferric reducing antioxidant power; TE, Trolox equivalent, DW, dry weight. Values are mean ± standard deviation (SD) of three repetitions.

## Data Availability

The data used to support the findings of this study are included in this article.
